# Ecological decoding of visual aesthetic preference with oscillatory electroencephalogram features—A mini-review

**DOI:** 10.3389/fnrgo.2024.1341790

**Published:** 2024-02-21

**Authors:** Marc Welter, Fabien Lotte

**Affiliations:** Inria Center at the University of Bordeaux/LaBRI, Talence, France

**Keywords:** Electroencephalography (EEG), brain-computer-interfaces, neuroaesthetics, aesthetic preference, oscillatory activity

## Abstract

In today's digital information age, human exposure to visual artifacts has reached an unprecedented quasi-omnipresence. Some of these cultural artifacts are elevated to the status of artworks which indicates a special appreciation of these objects. For many persons, the perception of such artworks coincides with aesthetic experiences (AE) that can positively affect health and wellbeing. AEs are composed of complex cognitive and affective mental and physiological states. More profound scientific understanding of the neural dynamics behind AEs would allow the development of passive Brain-Computer-Interfaces (BCI) that offer personalized art presentation to improve AE without the necessity of explicit user feedback. However, previous empirical research in visual neuroaesthetics predominantly investigated functional Magnetic Resonance Imaging and Event-Related-Potentials correlates of AE in unnaturalistic laboratory conditions which might not be the best features for practical neuroaesthetic BCIs. Furthermore, AE has, until recently, largely been framed as the experience of beauty or pleasantness. Yet, these concepts do not encompass all types of AE. Thus, the scope of these concepts is too narrow to allow personalized and optimal art experience across individuals and cultures. This narrative mini-review summarizes the state-of-the-art in oscillatory Electroencephalography (EEG) based visual neuroaesthetics and paints a road map toward the development of ecologically valid neuroaesthetic passive BCI systems that could optimize AEs, as well as their beneficial consequences. We detail reported oscillatory EEG correlates of AEs, as well as machine learning approaches to classify AE. We also highlight current limitations in neuroaesthetics and suggest future directions to improve EEG decoding of AE.

## 1 Introduction

Modern humans live in environments in which we are almost constantly confronted with artifacts. Many of these artifacts primarily serve instrumental purposes, i.e., they are designed to fulfill specific practical goals. Although, the design of many of these artifacts also take aesthetic considerations into account, a subset of artifacts are especially appreciated for their aesthetic qualities and designated with the label “art” or “artwork”. The field of neuroaesthetics studies the neural correlates of aesthetic experience (AE) (Vessel, 2022). While some of this research looks at aesthetics from the lens of economics and aims to discover sensory objects feature that increase economic interests such as product sales (Costa-Feito et al., 2019), an increasing number of studies report positive correlations between AEs and health and wellbeing (e.g., Fancourt and Finn, 2019; see Skov and Nadal, 2023, for a critique of such reports). With the internet and social media, many humans have now an unprecedented access to artworks. The abundance of available digital art together with advances in machine learning could allow for the personalization of AE with brain-computer-interfaces (BCI), and thus increase a kind of user experience that might positively affect human existence. BCIs are systems that allow direct communication between computers and brain signals (Vidal, 1973). For example, BCIs that decode correlates of AE could be used in combination with recommender systems that curate personalized art exhibitions in virtual museums in order to optimize visitor satisfaction. Although, the personalization of AE based on behavioral data already exists in digital spaces such as social media, such personalization algorithms often require explicit user feedback which can interrupt AE and thereby diminish user experience.

In order to alleviate this current limitation of AE personalization, the development of human-machine-interfaces that implicitly measure individual AE in real time offers a promising lead. One type of human-machine-interface that seems particularly suited to this endeavor are passive non-invasive BCI such as those based on Electroencephalography (EEG). Previous neuroimaging during AEs focused mostly on correlating beauty/pleasantness ratings with functional Magnetic Resonance Imaging (fMRI) signals (Vessel, 2022) and EEG Event-Related-Potentials (ERPs) (Jacobsen and Klein, 2022) in unecological lab conditions. While fMRI cannot be used in ecological conditions, EEG is used in many environments, even real museums (King and Parada, 2021). However, aesthetic preference does not necessarily arise time-locked to stimulus onset and might develop over larger timescales (Carbon, 2023). Thus, in addition to ERPs, EEG oscillatory features can contain information about aesthetic preference (Strijbosch et al., 2022). Therefore, EEG oscillatory features could be promising for aesthetic passive BCIs in ecological conditions outside the lab. However, the neuro-cognitive mechanisms of art experience in general and art preference in particular, remain unclear and more empirical research on neuro-aesthetics is required. This article summarizes the state-of-the-art in aesthetic preference decoding based on oscillatory EEG features and develops a road map toward the development of ecologically valid passive EEG-based BCI systems that could personalize user AE.

## 2 Aesthetic experience and art preference

AE can be defined as “a perceptual experience that is evaluative, affectively absorbing and engages comprehension (meaning) processes” (Vessel, 2022). This definition corresponds to the Aesthetic Triad Model (Chatterjee and Vartanian, 2014) which, based mostly on experimental aesthetics fMRI research, considers AE emerging from the interaction of three brain systems: sensory-motor, emotion-valuation, and knowledge-meaning. Similarly, Schaeffer (2015) postulates three main components of AE: attention, affect, and reward. To be precise, Schaeffer uses the French word “emotion” instead of “affect,” but following the definitory framework proposed by Schiller et al. (2023), we use the more general term “affect” composed of valence, arousal, and motivation. In this framework, “emotions” are considered a subset of conscious affects. Although, AEs are often related to the perception of artworks, the definitions above remains neutral with regards to the categorical status of the perceived objects, and it seems plausible that non-art objects, e.g., natural landscapes, can evoke similar AEs to art-related AEs. Nonetheless, the scope of this mini-review is constrained to the decoding of AEs during art watching which seems the most practical condition for improving AE with a BCI.

It seems evident that attentional information regulation is needed for any kind of experience. And indeed, works of art often invite attention. However, attentional EEG markers alone might not be ideal or sufficient features for AE decoding. Both negatively and positively valenced stimuli draw high amounts of attention due to the evolutionary significance of searching rewards and avoiding threats (Karim et al., 2017). Furthermore, both internally and externally oriented attentional mechanisms are involved in the processing of visual AE (Ansorge et al., 2022).

AEs cover a vast spectrum of different affective experiences (Menninghaus et al., 2018) which makes it very difficult to train a machine learning model to discriminate between all different classes of AE (Mühl et al., 2014). However, as we are primarily concerned with improving AE of art watching, we can simplify the problem to decoding and ranking aesthetic preference for various art stimuli.

Many neuroaesthetic fMRI studies reported activation of reward and pleasure processing areas in the brain during aesthetic experiences (Chuan-Peng et al., 2019). In the aesthetic literature, “pleasantness” often refers to objective stimulus features related to beauty (e.g., harmony, symmetry) (e.g., Babiloni et al., 2013). We, on the other hand, define “pleasantness” as related to an activation of hedonic brain systems that might be independent of objective stimulus properties. As Berridge and Kringelbach (2015) show, the reward system of the human brain consists of two major independent pathways, one related to pleasure and “liking” generated by opioids and endocannabinoids, and another one related to motivation and “wanting” produced by dopamine. Furthermore, activation of these reward circuits may not lead to the conscious experience of “liking” or “wanting” (Berridge and Winkielman, 2010). These types of experiences are difficult to decode, because training labels come from subjective aesthetic ratings. Therefore, preferring one aesthetic stimulus over another aesthetic stimulus is not reducible to feeling a higher amount of pleasure while perceiving this stimulus compared to the other one. Nor is beautiful art always liked more than non-beautiful art (Muth et al., 2020). The independence of these two reward systems has led scientists in the tradition of Kantian aesthetics (Kant, 1983) to affirm the nature of aesthetic appreciation as inherently disinterested (e.g Sarasso et al., 2020). Indeed, empirical data suggest that aesthetic pleasure does not need to be correlated with extrinsic motivations such as the desire to own or control the appreciated aesthetic object (Chatterjee and Vartanian, 2016). However, from this it does not follow that aesthetic appreciation is essentially disinterested, because even though the aesthetic object might not be perceived in an instrumental fashion to fulfill an external goal, beholders might be intrinsically motivated and therefore interested to interact with an aesthetic object, because they will experience intrinsic rewards through this interaction. It has been shown that motor activity can be suppressed during AEs such as the perception of beauty (Kawabata and Zeki, 2004). When Sarasso et al. (2020) interpret this phenomenon as evidence for aesthetic disinterest, they miss that movement is often a constitutional part of AE and that moments of stillness can lead to further motor interaction with an artwork (Kühnapfel et al., 2023). Because AEs are intrinsically rewarding, they motivate to prolong the AE and to search for more AEs in the future (Reeves, 1989). Still, the exact role of rewards in AE remains controversial. Some scientists claim that aesthetic appreciation can be reduced to the valuation of sensory objects (Skov and Nadal, 2020), whereas others disagree with this reduction (Vessel, 2022). Nonetheless, we hypothesize that EEG correlates related to reward processing should be discriminative for aesthetic preferences.

## 3 Oscillatory EEG correlates of visual aesthetic preference

As many Machine Learning classification algorithms require hand-crafted features, the following section will describe oscillatory EEG correlates of visual aesthetic preference that could constitute informative features. We conducted a literature review by searching public databases, as well as references in the neuroaesthetic literature. The search query was: “+aesthetic*|art*|paint*+*EEG*|*brain*|neur*+oscillat*|wav*|frequen*|*rhythm*.” We only included studies reporting oscillatory EEG correlates of AE for static visual art stimuli in naturalistic conditions. These findings are discussed as potential correlates of attention, affect and reward during AE (see Table 1 for a summary).

**Table 1:** This table summarizes EEG rhythm modulations correlated to attentional, affective and reward components of AE.

**EEG markers**	**Attention**	**Affect**	**Reward**	**Modulation**	**Mental correlates**
Frontal alpha asymmetry		x	x	↑	Motivation (Wacker et al., 2013) Pleasure (Babiloni et al., 2013)
Occipital/parietal alpha	x			↓	Attention (Peylo et al., 2021) DMN (Vessel et al., 2012) Pleasure (Rawls et al., 2021)
Frontal beta	x	x		↓	Engagement (Herrera-Arcos et al., 2017) Empathy (Schubring and Schupp, 2019)
Centroparietal gamma	x	x	x	↑	Savoring (Strijbosch et al., 2022)

“x” denotes hypothesized links between EEG markers and components of AE, whereas “↑” and “↓” correspond to the directions of oscillatory modulations, i.e., “↑” indicates that this EEG marker's value increases for preferred artworks. The last column lists possible mental correlates related to these modulations.

### 3.1 Attention

Although correlates of visual attention are widely studied with EEG and harnessed in BCI (Nam et al., 2018), to our knowledge only Rawls et al. (2021) report rhythm modulations commonly associated with visual attention that were informative of art preference. Instructing 44 subjects to give preference ratings to binarized Jackson Pollock paintings and computer generated cantor fractals after 4s viewing time, they found a suppression of the parietal alpha rhythm correlated with art preference. Parietal alpha modulations have been implicated in attentional processing (Peylo et al., 2021) and the Default Mode Network (DMN) which has been linked to aesthetically moving art watching (Vessel et al., 2012, 2013). However, the authors noted that it might be related to stimulus properties such as visual complexity and not necessarily be related to preference. Another brain rhythm associated with the DMN, the theta rhythm, was investigated by Strijbosch et al. (2022) during aesthetically moving art watching, but they found no relation between theta modulations and AE.

### 3.2 Affect

Many of the brain areas involved in affective processing are located subcortically which makes it difficult to measure their activity with EEG (Mühl et al., 2014). Nonetheless, beta suppression has been reported during empathy and affective processing in general (Schubring and Schupp, 2019). Herrera-Arcos et al. (2017) conducted a mobile EEG study (#subjects = 25) during an Otto Dix exhibition in a real museum with a commercial Muse EEG. Their analyses show a correlation between beta suppression and artwork preference. This beta suppression was interpreted as related to emotional engagement by the authors. However, it is also possible that the measured beta suppression was generated by motor activity, especially under mobile recording conditions (Pope et al., 2022). Still, motor activity could potentially be related to affective processing and informative of aesthetic preference (Kühnapfel et al., 2023).

### 3.3 Reward

fMRI scans during AEs often report activation of brain areas involved in reward processing (e.g., Kawabata and Zeki, 2004). Unfortunately, similarly to affective processing, many reward related areas in the brain are also subcortical and hard to measure. Still, reward related-signals in the frontal cortex could be measurable by frontal EEG electrode, an hypothesis which is strengthened by a multitude of experimental reports of a relation between frontal alpha asymmetry (FAA) in the EEG and reward processing across different reward modalities (see Sabu et al., 2022 for a review). Although, FAA has been linked to pleasure and liking, some suggest that it is generated by motivational dopamine release (Wacker et al., 2013). Babiloni et al. (2013) collected mobile EEG data (#subjects = 25) during a Dutch Golden Age exhibition in a museum and found a correlation between FAA and beauty/pleasantness ratings. Babiloni et al. (2015) reproduced this correlation during a Titian exhibition (#subjects = 27). Cheung et al. (2019) also reported a link between FAA and beauty ratings (#subjects = 20) for Western art displayed on a screen. Therefore, FAA could be a good marker for an AE that motivates to prolong the AE or to reproduce it in the future. However, one has to keep in mind that FAA is related to motivational processing in general and not limited to a particular valence (Lacey and Gable, 2022). Thus, artworks that evoke anger or other negatively valenced approach-motivation related experiences could also generate FAA.

### 3.4 Mixed component

Strijbosch et al. (2022) studied the neural dynamics during aesthetically moving experiences with EEG. Showing 35 participants a wide range of diverse artworks for 6 s, they reported a gamma increase 1 s after stimulus presentation until the end of the trial. This was interpreted as a correlate of savoring, a process of up-regulation of positive affect by sustaining attention on an experience. Similar gamma modulations were found with regards to other types of positively affecting AE, such as enjoying the taste of chocolate (Berk et al., 2016; Silver et al., 2018). EEG gamma rhythms are often contaminated by muscle artifacts and are, therefore, commonly filtered out in EEG analysis (Jeunet et al., 2018). And although, Strijbosch et al. (2022) followed a rigorous artifact removal protocol, muscle contamination remains possible.

Now that we have reviewed potential oscillatory EEG markers of aesthetic preference, we will discuss their use as features for machine learning classification of AE.

## 4 Classification of aesthetic preference based on oscillatory EEG markers

Almost no publications reporting aesthetic preference classification results based on EEG signals exist to our knowledge. Fraiwan et al. (2023) reported an aesthetic enjoyment classification accuracy above 98% using a deep neural network and Multiscale Entropy features on a mobile data set from Cruz-Garza et al. (2017) (#subjects = 28). EEG Entropy measure have been shown to contain meaningful information for emotion decoding (Patel et al., 2021) and decoding of aesthetical preference of music (Carpentier et al., 2019). However, EEG classification almost never yields such high performance in practice and we should remain skeptical (Jeunet et al., 2018). The data could contain informative muscular artifacts, as the authors do not report any artifact removal protocol or the trained model could have been overfitted on the data, as deep learning models often are (Goodfellow et al., 2016). Nonetheless, brain entropy measures could be useful for visual aesthetic preference decoding.

Mazzacane et al. (2023) used a novel classification algorithm based on temporal decision trees (Sciavicco and Stan, 2020) to extract symbolic rules ralating EEG amplitudes of specific frequency bands and electrode locations to aesthetic liking or disliking in an ecological museum context (#subjects = 16). The authors report high classification performance using features from beta and gamma bands that could be related to affective and reward processing. However, we remain skeptical to their claim that their temporal decision tree classification algorithm makes muscle artifact removal unnecessary and hypothesize that the beta and gamma activity used by the classifier could be generated by muscular and movement artifacts since they were not controlled for, cleaned nor removed.

Surprisingly, more established EEG classification algorithms (see Lotte et al., 2018, for a review) have not been applied to AE decoding to our knowledge, and should be explored in the neuroaesthetic domain. Deep learning classifiers (e.g., Lawhern et al., 2016; Schirrmeister et al., 2017) might be worth investigating as well, because our theoretical and empirical knowledge about which EEG features contain discriminant information for AE decoding is lacking. However, large public datasets required to benchmark decoding algorithms of AE are missing (Jayaram and Barachant, 2018). For now, BCI classification algorithms based on Riemannian Geometry seem to work best and often outperform deep learning methods (Congedo et al., 2017; Roy et al., 2022) which is why some state-of-the-art EEG BCI decoding implements Riemmanian Geometry in deep neural network architectures (e.g., Kobler et al., 2022).

## 5 Discussion

Our literature search yielded six publications that investigated oscillatory EEG markers of AE that reported EEG modulations in alpha, beta and gamma frequency bands. This variability could be explainable by different experimental protocols, e.g., artworks used and aesthetic ratings. Thus, robust EEG features to decode aesthetic preference that generalize to all types of AE remain unclear.

### 5.1 Pitfalls

We related specific EEG frequency bands with attentional, affective and reward-related components of AE, however neural rhythms can not be one-to-one mapped to mental states (Brouwer et al., 2015). Furthermore, EEG measurements suffer from high variability within and between subjects and even data from the same subject might be very different if recorded at different times (Fairclough and Lotte, 2020). Additionally, it has been shown that context influences AE, and AEs in laboratory conditions are quite different from AE in the wild (Carbon, 2020). As such, we do not know whether correlates of AE discovered during a laboratory experiment will generalize to natural contexts outside the laboratory. To our knowledge, only FAA has been reported in both conditions. However, in experimental conditions, even in real museum settings, subjects often have to look at an affective stimulus, even if they would prefer not to, which influences FAA (Lacey and Gable, 2022).

Due to the need of a preference groundtruth, and without direct access to reward processing information in the brain, aesthetic preference decoding has to rely on explicit rating tasks that assign a subjective value to an artwork. This explicit aesthetic judgment task can introduce confounds, as shown by ERPs that only appear with aesthetic rating tasks (Höfel and Jacobsen, 2017). Similarly, some EEG oscillations might be related to aesthetic judgment and not AE *per se*. Therefore, these might not appear in natural art watching conditions.

Different artworks might evoke very different AEs and consequently different EEG signals. Additionally, various visual stimulus features are known to affect the EEG signal such as luminance (Eroğlu et al., 2020) or complexity (Rawls et al., 2021). Therefore, it remains unclear, whether empirical results gathered with one type of art stimuli will generalize to other types of art. Last, but not least, natural visual AE is an embodied experience that involves motor processes such as eye movements. Therefore, EEG data of such experiences should be subjected to similar rigorous artifact removal protocols as other mobile EEG imaging scenarios (Gorjan et al., 2022).

### 5.2 Future work

It was mentioned before that the subcortical location of many reward and affect processing areas involved in aesthetic processing makes EEG decoding difficult. Nonetheless, their activity can be estimated using computational modeling. Singer et al. (2023) used fMRI-informed EEG models of reward processing to decode activity from the ventral striatum using EEG and demonstrated good model performance across aesthetic and non aesthetic domains, as well as across subjects. Another possibility that could improve decoding of aesthetic appreciation would be to use EEG source localization algorithms with realistic headmodels and define reward areas as regions of interest. Although EEG source localization is limited in its accuracy, empirical results have shown that extracting information from source localized regions of interest can improve classification performance (Edelman et al., 2019). One limitation of this approach is that source localization algorithms are computationally expensive. Potentially, investigating functional connectivity of EEG electrodes (Gonzalez-Astudillo et al., 2022), might be an alternative and/or a complement to anatomical source space feature extraction. Brain networks such as the DMN are activated during AE (Vessel, 2022). Kontson et al. (2015) reported increased functional connectivity between frontal and parieto-occipital EEG electrodes during art watching compared to a baseline. However, they did not investigate functional connectivity for different aesthetic preference levels.

Finally, AE is not exhausted by attention, affect, and reward, but also includes semantic and motor processes. While decoding semantic content in EEG signals is very difficult (e.g., Gifford et al., 2022), EEG motor correlates are relatively well studied in BCI (Yuan and He, 2014). Neuroaesthetic research has shown that art watching can activate motor related brain areas which could be related to motor simulation (e.g., Umiltá et al., 2012) and empathy (Gallese, 2017). Unfortunately, the link between embodied cognition and art appreciation has not been shown using neuroimaging. However, correlations between motor priming and art appreciation have been reported (e.g., Ticini et al., 2014). We hypothesize that EEG correlates of motor control such as mu rhythm modulation could, therefore, be informative of AE.

## 6 Concluding remarks

Neuroaesthetic research on AE decoding is still in its infancy. We reviewed sparse and inconsistent reports of EEG oscillatory correlates of AE. Although, we focused on AE of visual art here, empirical data suggests that other AEs are not fundamentally different (Vessel, 2022). Thus, some of the neural correlates of art preference reviewed here could generalize to other types of preference. Still, our focus on EEG for AE decoding constitutes a limitation. Other physiological signals, e.g., heart rate and skin conductance, are informative of affective states in general (Shu et al., 2018; Rinella et al., 2022) and of AE in particular (Kühnapfel et al., 2023). Indeed, combining EEG with these signals can improve mental state classification (Hogervorst et al., 2014) and should be explored for AE decoding. Overall, there remain a number of challenges to solve before BCI could reliably and rigorously decode AE from EEG, for different artworks and different contexts. We hope this mini-review modestly contributed to identify these challenges and to propose relevant directions for future works.

## Author contributions

MW: Conceptualization, Data curation, Investigation, Methodology, Writing — original draft. FL: Conceptualization, Funding acquisition, Methodology, Project administration, Supervision, Writing — review & editing.
